# Distinctive fish collagen drives vascular regeneration by polarizing macrophages to M2 phenotype via TNF-α/NF-κB pathway

**DOI:** 10.1016/j.mtbio.2025.102273

**Published:** 2025-09-03

**Authors:** Yuanchi Wang, Honghui Jiang, Yiping Wang, Yifan Wu, Xixi Wang, Ju Zhang, Yeqi Nian, Jing Liu, Zhihong Wang

**Affiliations:** a Tianjin Key Laboratory of Biomaterial Research, Institute of Biomedical Engineering, Chinese Academy of Medical Sciences and Peking Union Medical College, Tianjin, 300192, China; b Institute of Transplant Medicine, School of Medicine, Key Laboratory of Bioactive Materials of Ministry of Education, Nankai University, Tianjin, 300071, China; c College of Life Sciences, Tiangong University, Tianjin, 300387, China

**Keywords:** Collagen, Fish swim bladder, Macrophage polarization, Small diameter vascular graft

## Abstract

Collagen is a structural protein that plays a critical role in tissue regeneration and is widely utilized in biomedical applications. Recent studies have demonstrated that collagen can modulate macrophage polarization; however, most studies have focused on mammalian collagen such as type I collagen derived from bovine and pig sources. In this study, we performed a comprehensive investigation of the role of collagen derived from aquatic sources, specifically fish swim bladder-derived collagen (SCC), in modulating macrophage inflammation using *in vitro* and *in vivo* experiments. First, collagen-coated and collagen-incorporated electrospun poly(ε-caprolactone) (PCL) films were prepared. RNA-Seq analysis showed that SCC could promote M0 and M1 phenotype macrophage transition into M2 through the activation of TNF-α/NF-κB and the downstream signaling pathways. Subcutaneous implantation and artery replacement were also performed. Moreover, SCC prolonged coagulation and synergistically reduces the risk of stenosis. Finally, mouse carotid artery replacement demonstrated that the SCC-modified vascular graft exhibited higher patency in combination with rapid endothelialization and reduced inflammatory responses *in vivo*. Taken together, we provide strong evidence that fish swim bladder-derived collagen has the capability to modulate macrophage polarization and shows great potential for tissue remodeling and regeneration.

## Highlights

• SCC could induce macrophage polarization via TNF-α/NF-κB and its downstream pathways.
• SCC could enhance the patency and regeneration of vascular grafts in a mouse carotid artery model.
• SCC may serve as an immunomodulatory agent for anti-inflammatory responses.

## Graphical abstract

**Figure:**
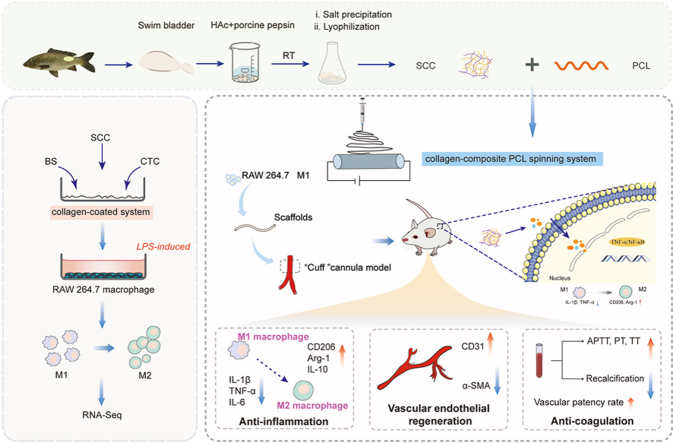

## 1 Introduction

Collagen, a key structural protein in the extracellular matrix, plays a crucial role in supporting and repairing tissue cells. In addition to its structural function, collagen exerts significant biological effects *in vivo* through its ability to regulate diverse cellular processes such as cell adhesion, migration and signal transduction [[1], [2], [3], [4], [5]]. Owing to its ideal characteristics for use as a biomedical material, collagen has been widely adopted in various medical applications, such as medical dressings, bone repair and biological scaffolds [[5], [6], [7]]. Collagen is mainly derived from animals such as mammals and fish, and the preparation of collagen from animal sources is a relatively straightforward process, typically involving the use of acid, salt or alkali methods [8]. Enzyme treatment methods have been proven to reduce immunogenicity and increase the yield. Furthermore, recombinant collagen, synthesized using genetic engineering techniques, represents a new source of collagen [9,10]. However, their weak thermal stability and susceptibility to enzymatic degradation require further optimization. A notable challenge pertains to the low hydroxyproline hydroxylation rate of recombinant collagen, which hinders its ability to form a stable triple-helix structure. Synthetic collagen [11] is synthesized by creating a trimeric structure of Gly-X-Y repeats and similar peptides in the primary peptide chain structure of collagen, which has attracted significant attention but is currently costly to produce and requires further verification in terms of safety and efficacy. In contrast, collagen obtained from animal sources retains its natural triple-helical structure, and its biological effects are preserved. Consequently, it is still the most important resource for existing research fields and applications in biomedicine.

Fish collagen offers superior bioavailability, lower risk of zoonotic diseases and better biocompatibility than mammalian or recombinant collagen, making it safer and more effective for biomedical applications [12]. However, collagen extracted from fish skin, bones and fins exhibits a low thermal denaturation temperature (Td), which commonly ranged from 25 °C to 35 °C [13], making it susceptible to inactivation at body temperature. Interestingly, a recent study reported that the denaturation temperature varied for collagen from different organs, and the internal organs showed a higher Td than the superficial tissue. In line with this, our previous study found that the Td of swim bladder collagen (SCC) can reach 40.08 °C [14,15], proving that SCC is more stable. SCC is a highly pure type I collagen with a well-preserved triple-helical structure, which maintains the same characteristics as mammals. The collagen from fish bladders is specifically rich in cysteine, which is not detected in bovine pericardium. The decellularized extracellular matrix (dECM) of fish bladders, especially the collagen, have higher contents of threonine, methionine, isoleucine and phenylalanine than those from bovine pericardium [14]. Besides, a comparison analysis of collagen from mammalian and fish swim bladder revealed significant disparities in electrophoretic profile, peptide profile, amino acid composition and biochemical properties [15,16]. For instance, the unique cysteine was present in swim bladder collagen, which has been shown to self-assemble into delicate and dense fibers under specific conditions, possess a higher antioxidant capacity [14]. Based on this, we can speculate that swim bladder collagen may have some unique biological effects.

Macrophage polarization plays a crucial role in regenerative medicine and tissue engineering by modulating inflammation and tissue repair during wound healing [[17], [18], [19]]. Macrophages are categorized into two main phenotypes, M1 (pro-inflammatory) and M2 (anti-inflammatory), based on their functional state [20]. The balance between these phenotypes is critical for tissue regeneration and healing outcomes [21]. M1-type macrophages initiate an immune response through the secretion of pro-inflammatory factors (e.g., TNF-α and IL-1β). However, if unregulated, this can lead to excessive inflammation. M2-type macrophages, in turn, secrete anti-inflammatory factors (e.g., including IL-10 and TGF-β) that reduce inflammation, promote tissue repair and stimulate collagen production. Various strategies for regulating macrophage polarization are evolving rapidly in regenerative medicine [[22], [23], [24]]. RAW264.7 is a commonly used model cell line as the typical macrophage in studying macrophage polarization. Recent studies have investigated the effects of biomaterials, small molecules, drugs, cellular therapies and their combinations on macrophage function [25,26]. The tunability of biomaterials, such as surface properties, mechanical properties and dynamic behavior, has been demonstrated to be a highly effective strategy. Recently, collagen has been reported to influence macrophage polarization attributed to its properties [27]. Hao et al. [28] reported that collagen extracted from porcine bone can inhibit pro-inflammation by decreasing the release of IL-6 and TNF-α in LPS-induced RAW264.7 cells. In addition, Alves AL et al. [29] introduced collagen from shark skin exhibiting low immunogenicity, which induced low expression of pro-inflammatory cytokines and high expression of Arg1. An in-depth study of the effects of swim bladder collagen on macrophage polarization is important for future biomedical applications.

**Scheme 1:**
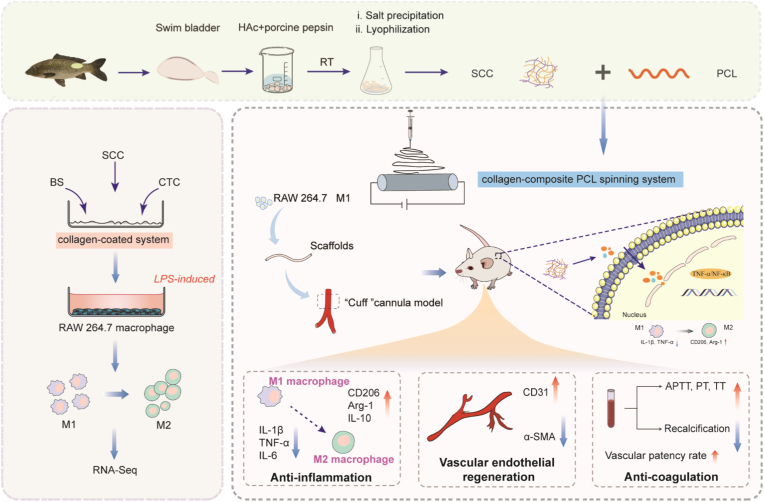
Schematic illustration of study design and macrophage polarization mediated by SCC.

Therefore, this study focused on investigating the immunomodulatory effects of fish swim bladder collagen, specifically on macrophage polarization (Scheme 1). Initially, two types of valid systems, comprising collagen coating and collagen incorporation into polycaprolactone (PCL) spinning scaffolds, were developed. The effect of SCC on macrophage polarization and the mechanisms underlying these processes were investigated by *in vitro* assays, including RNA-Seq analysis. Furthermore, a type of small-diameter vascular graft based on PCL/SCC was prepared, and its performance *in vivo* was examined by a mouse carotid artery graft model, as well as its function, inflammation modulation and tissue regeneration. Overall, this study demonstrated that SCC can regulate macrophage polarization from M1 to M2 through the activation of TNF-α/NF-κB and its downstream signaling pathways.

## 2 Materials and methods

### 2.1 Preparation and characterization of SCC

Fresh silver carp (*Hypophthalmichthys molitrix*) fish swim bladders (Yulong Fishery, Wuhan, China) were adopted preferably [30], cleaned, cut and treated with 0.15 M NaOH, distilled water and 10 % isopropyl alcohol. Collagen was extracted by soaking in 0.5 M HAc with 0.2 % porcine pepsin for 48 h. The supernatant was centrifuged (10,000 rpm, 4 °C, 15 min), salted with NaCl (up to 2.5 M) and centrifuged again after 12 h. The precipitate was dissolved in 0.5 M HAc, dialyzed in 0.02 M HAc for 48 h, lyophilized and stored at −20 °C. SDS-PAGE was used to determine the molecular weight. UV spectra (Lambda 35-PerkinElmer, USA) and FTIR (Thermo FT-IR spectrometer Nicolet IS10, USA) were also performed.

### 2.2 Collagen coating

Dissolve the collagen SCC and collagen from the cow calcaneal tendon (CTC, type I, McLean, China) in 0.5 M HAc to make 0.1 mg/mL collagen solution. Collagen at a concentration of 10 μg/cm^2^ was applied to the bottom of the 6-well plate and incubated at 37 °C for 2 h. The collagen solution in the well plate was discarded, washed three times with PBS and stored at 4 °C. To verify the surface morphology, the micromorphology was observed using scanning electron microscopy (SEM, Hitachi SU5000, Japan). In addition, the roughness of the coating surface was investigated using atomic force microscopy (AFM, Bruker, Germany).

### 2.3 Macrophages culture on collagen coating surface *in vitro*

RAW264.7 macrophages from Peking Union Medical College (China) were induced to M1 type with 100 ng/mL LPS (Sigma, USA), and a non-induced control group was set up. Macrophages were seeded at 1 × 10^6^ cells per well in SCC, Blank substrate (BS) and CTC collagen-coated 6-well plates. After 24 h of culture, the cells were collected for quantitative real-time polymerase chain reaction (qPCR) to detect *IL-6, IL-1β, TNF-α, CD206, Arg-1 and VEGF* gene expression. Using β-actin as the reference gene, the relative gene expression was analyzed using the 2^−ΔΔCt^ method. All primers used are listed in Supplementary Table S1 (Sangon, China).

For immunofluorescent staining, cells were fixed with 4 % paraformaldehyde for 20 min at room temperature (RT), permeabilized with 0.1 % Triton X-100 for 10 min, incubated with primary antibodies against iNOS (1:200, ab283655, Abcam, UK) and CD206 (1:200, ab300621, Abcam, UK) at 4 °C overnight, then with the secondary antibody (1:100, ab150080, Abcam, UK) for 2 h, and finally with DAPI for 10 min. Images were captured using an inverted fluorescence microscope (Leica, Germany).

### 2.4 Fabrication of electrospinning scaffolds

PCL (Mn = 80,000) (Sigma, USA) and SCC were dissolved in Hexafluoroisopropanol (HFIP, McLean, China) with a PCL/SCC ratio of 9:1 to obtain a final concentration of 15 % (w/v), and were then magnetically stirred overnight at RT with a 21G blunt-ended needle and set at a constant flow rate of 2 mL/h. A voltage of 18 kV was applied between the needle and the cylinder collector. The rotation speed of the collector was 500 rpm, and the distance between the needle and the collector was 15 cm. Then, to prepare the tubular scaffolds, the collector was replaced with a stick with an inner diameter of 7 mm using the same spin parameters, and the surface morphology of the PCL/SCC scaffolds was observed with an SEM (Hitachi SU5000, Japan). X-ray photoelectron spectroscopy (XPS) was carried out using an X-ray photoelectron spectrometer (ESCALAB Xi+™, Thermo Fisher Scientific) in the range of 0–1350 eV and at an angle of 90°. The nanofiber diameters and pore sizes of the electrospinning scaffolds were measured using Image J by randomly choosing 100 fibers from five SEM images.

### 2.5 Cell culture and cytocompatibility

Purified murine RAW264.7 and L929 cells from Peking Union Medical Collage (China) were cultured in high-glucose DMEM (Gibco, USA) with 10 % FBS (Gibco, USA) and 1 % penicillin/streptomycin (Solarbio, China) at 37 °C in 5 % CO_2_. Human Umbilical Vein Endothelial Cells (HUVECs) from the same source were cultured in ECM (ScienCell, USA) with 10 % FBS, 1 % penicillin/streptomycin and 1 % ECGS under the same conditions. The biocompatibility tests included cytotoxicity, cell adhesion and live/dead staining. Membrane materials were incubated with high-glucose DMEM at 6 cm^2^/mL for 24 h at 37 °C to prepare the extract, which was filtered through a 0.22 μm microporous membrane. HUVECs and L929 cells were cultured with 200 μL of extract, and OD values were measured at 450 nm using a cell counting kit-8 (CCK-8 kit, Meilun Biology, China) at 24, 48 and 72 h. For live/dead staining, after 24 h of co-culture with cells (1 × 10^5^/well), the working solution was added and incubated at 37 °C for 40 min. HUVECs (5 × 10^4^ per dish) were seeded on materials and cultured for 24 h and 72 h, then fixed with 4 % paraformaldehyde for 20 min, permeabilized with 0.1 % Triton X-100 for 10 min, incubated with 5 g/mL fluorescein Isothiocyanate (FITC, Solarbio, China) for 1 h at RT, incubated with 4′,6-diamidino-2-phenylindole (DAPI, Beyotime, China) for 10 min and imaged by a laser confocal microscope (Nikon AX, Japan).

### 2.6 Hemocompatibility test *in vitro*

All animal experimental procedures were approved by the medical ethics committee and performed in accordance with the guidelines for the care and use of laboratory animals of the Peking Union Medical College (China). Sprague-Dawley (SD) rats from Vital River (China) were used in this study. The animal ethics permit number is IRM-DWLL-2022014, and the implementation period of the animal ethics permit is from January 2023 to December 2026. Platelet-rich plasma (PRP, 150 g, 15 min) and platelet-poor plasma (PPP, 1500 g, 15 min) were obtained. The samples in the 24-well plates were rinsed three times with saline. Next, 400 μL of PPP was added to each well and incubated at 37 °C for 2 h. Activated partial thromboplastin time (APTT), prothrombin time (PT) and thrombin time (TT) were measured using an *in vitro* clotting time test kit (Shanghai Sun Biotech, China).

For the plasma recalcification experiment, 100 μL incubated PPP and 100 μL 0.025 M CaCl_2_ solution were added to each 96-well plate well, with a control group containing only PBS. The absorbance at 450 nm was measured every 30 s until it plateaued. A S-shaped absorbance-time curve was plotted. The inflection point on the recalcification curve was analyzed using Origin software by fitting the logistic equation. Following F.J. Richards et al. [31] defined the lag time (where f′′(x) = 0) to evaluate the anticoagulant ability of the material. The overall coagulation potential (area under the curve) was calculated for coagulation curve analysis as described by Zong et al. [32]. The Logistic curve density function is defined as follows:

$$f\left(x\right)=\frac{e^{-x}}{{\left(1+e^{-x}\right)}^{2}}$$

To determine the level of material to adhere to platelets, after incubating PRP with the material at 37 °C, samples were then immersed in a 2.5 % glutaraldehyde solution (Solarbio, China) specifically for electron microscopy (500 μL/well) and stored overnight at 4 °C. After three washes with PBS, they were sequentially dehydrated using an ethanol gradient for 10 min each time. Platelet adhesion was observed by SEM after lyophilization. The absorbance at 490 nm was measured using lactate dehydrogenase assay kit (LDH kit, Beyotime, China) to quantify platelet adhesion. Hemolysis of the material was also the subject of the test. After centrifugation, the supernatants of each group were added to a 96-well plate and the absorbance at 545 nm was measured using an ultraviolet spectrophotometer (Hitachi UH5700, Japan). The positive control was distilled water and the negative control was saline. The hemolysis rate was calculated using the following formula:

$$Hemolysis\;ratio\left(\%\right)=\frac{A0-A2}{A1-A2}*100\%$$

where A0 represents the absorbance of the experimental groups, A1 is the positive control, and A2 is the negative control.

### 2.7 Subcutaneously implantation *in vivo*

SD rats had food withheld for 4 h prior to the surgical procedure, with free access to water maintained until anesthesia induction. The SD rats were placed in the induction box and exposed to 4 % isoflurane + 1–2 L/min oxygen [33,34]. After induction, they were moved to the operating table and connected to a face mask to maintain anesthesia (1.5–2 % isoflurane). The hair on the back was removed, iodophor was applied for disinfection, and then the skin on the back was cut open for the operation. During the experiment, the respiratory rate and toe reflexes were monitored in real time. PCL and PCL/SCC membrane materials (1 × 1 cm in area and 250 μm in thickness) were subcutaneously implanted into SD rats (n = 3). After implantation for 1 and 4 weeks, the samples were fixed with 4 % paraformaldehyde, dehydrated with 50 % sucrose solution and embedded in Optimal Cutting Temperature Compound (OCT, SAKURA, USA). The frozen section thickness was 6 μm using a freezing microtome (Leica, Germany), and hematoxylin-eosin (H&E) staining was performed. Immunofluorescence staining of iNOS (1:200, ab283655, Abcam, UK), CD68 (1:200, ab955, Abcam, UK) and CD206 (1:200, ab300621, Abcam, UK) was performed to identify the macrophage phenotypes. All fluorescence images were obtained by confocal laser scanning microscopy (CLSM) and analyzed using Image J. In the *in vitro* test experiment section, the scaffold was co-incubated with RAW264.7 cells. Supernatants were collected and centrifuged, then cytokines *Arg-1, IL-10, IL-6 and TNF-α* in the supernatants were measured using ELISA kits (Enzyme linked organisms, Shanghai, China) according to the manufacturer instructions.

### 2.8 Vascular stents implantation *in vivo*

The polarized M1 cell suspension (3 × 10^7^/mL) was injected evenly from both ends along the vessel. The vascular stents were then loaded with the cells in the incubator for 2 h. The procedure of carotid vessel harvesting and the protocols as follows [35]: C57 mice were weighed, anesthetized with 4 % chloral hydrate, and the neck was depilated [36]. A longitudinal incision was made, muscles ligated with 5-0 sutures, and the right common carotid artery exposed. The artery was doubly-ligated with 9-0 sutures, transected, and a cuff secured with an arterial clamp. The vessel was everted over the cuff and sutured with 9-0. An artificial vessel was then placed over the everted segment and similarly secured. After removing clamps (proximal first), patency was confirmed via heparinized saline flush. The wound was closed with 6-0 sutures, and mice recovered in a warm environment. One month later, following re-anesthesia, the implant was explanted to observe the patency of the blood vessels, and were photographed and recorded using a stereomicroscope (Leica, Germany). Frozen tissue sections (6 μm) were prepared from the excised blood vessels using a frozen slicing machine (Leica, Germany) for H&E and immunofluorescence staining. For immunofluorescence staining, primary antibodies including α-SMA (1:100, ab314895, Abcam, UK), CD31 (1:70, ab222783, Abcam, UK), iNOS (1:50, ab283655, Abcam, UK) and CD206 (1:200, ab300621, Abcam, UK) were added and then left overnight at 4 °C. After returning to RT for 1 h, the secondary antibodies (ab150080, ab150077, Abcam, UK) were added for 2 h, then added with sealed tablets containing DAPI (Solarbio, China) and stored at 4 °C. All images were captured under an upright microscope (Zeiss, Germany) and statistical analysis was performed using Image J software.

### 2.9 RNA sequencing

RNA sequencing was performed to explore the possible expression of pro-inflammatory and anti-inflammatory factors. SCC and SCC coatings induced by LPS were also prepared. Blank substrate was used as the control group in the same manner. After M0 macrophages were cultured on the coatings for 24 h, total RNA was extracted using the Trizol reagent (Invitrogen, USA). RNA-Seq was performed using Megi Biology software (Shanghai, China). The GO (Gene Ontology), KEGG (Kyoto Encyclopedia of Genes and Genomes) pathway enrichment analyses and GSEA (Gene Set Enrichment Analysis) were further performed.

### 2.10 Statistical analysis

Quantitative experiments were repeated at least three times. Data are expressed as means ± standard deviation (SD). The *t*-test and one-way analysis of variance (ANOVA) from GraphPad Prism software (GraphPad, USA) was used to compare the differences. NS p > 0.05 was considered no significant difference, p < 0.05 was considered statistically significant, i.e., ∗p < 0.05, ∗∗p < 0.01, ∗∗∗p < 0.001 and ∗∗∗∗p < 0.0001.

## 3 Results and discussion

### 3.1 Preparation and characterization of SCC

**Fig. 1:**
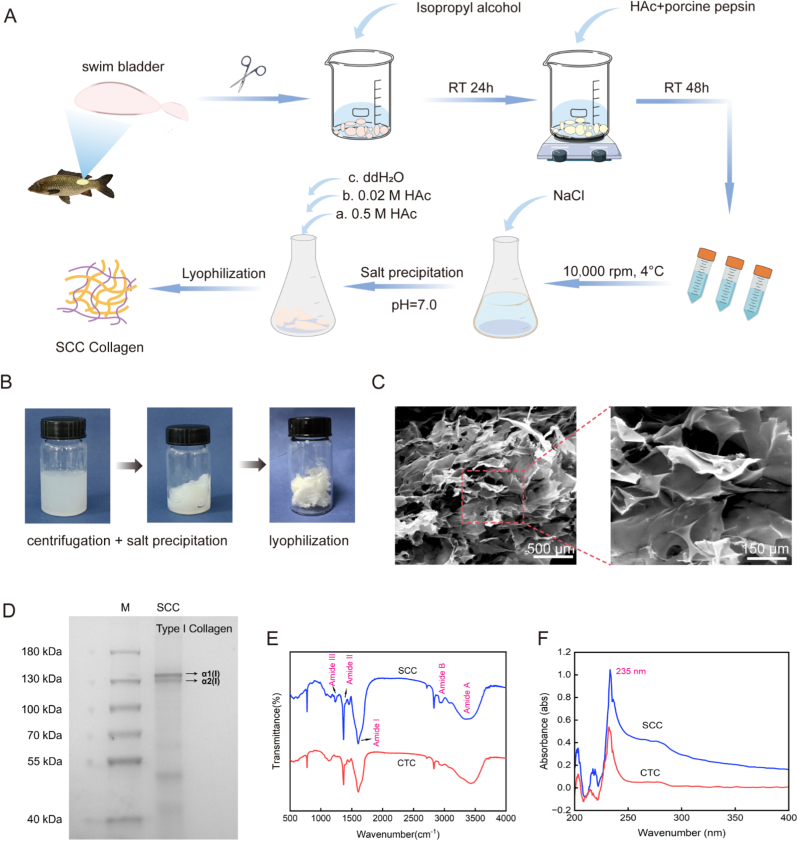
Schematic diagram and characterization of SCC extraction. (A). Schematic diagram of collagen extraction from the swim bladder of silver carp. (B). Key steps of SCC extraction include centrifugation, salting out, and freeze-drying. (C). SEM images of SCC, scale bar = 500 μm. (D). SDS-PAGE diagram. M is a protein marker. (E). Infrared absorption spectra of collagen. CTC represents cow calcaneal tendon bone collagen. (F). UV absorption spectrogram of collagen (absorption wavelength 200–400 nm).

SCC protein was extracted from fish swim bladders using acid extraction method. The process was provided as shown in Fig. 1A, and the SCC protein was obtained after centrifugation, salting out and freeze-drying (Fig. 1B). The morphology of SCC was a porous flocculent structure, as observed by SEM (Fig. 1C). SDS-PAGE revealed that it was type I collagen (Fig. 1D), which was mainly characterized by two chains, α1(I) and α2(I). It was inferred that the method of collagen extraction in this study did not result in the degradation of collagen, and it retained an intact molecular structure. Furthermore, the molecular weight was approximately 130 kDa, which is consistent with the reported in the literature [14]. Furthermore, the infrared spectra demonstrated that both the extracted collagen and commercially available collagen possessed the characteristic infrared absorption bands of type I collagen, indicating their respective compositions of amide A, B and amide I, II and III bands (Fig. 1E). The amide A band is typically located within the range of 3310-3270 cm^−1^. The presence of the amide B band, which is primarily attributable to the stretching vibration of the N-H, is indicated by 2900 cm^−1^ peaks. The amide I band is frequently observed in the vicinity of the wavelength of 1600 cm^−1^, which primarily reflects the C=O stretching vibration of the protein, thereby effectively reflecting the existence of the triple helix structure of collagen. The amide II band is typically located within the 1580-1500 cm^−1^ wavelength interval, indicative of the N-H bending vibration of the protein. The presence of the amide III band is indicative of the bending vibration of N-H and stretching vibration of C-N. The UV absorption spectra of SCC and CTC (cow calcaneal tendon bone collagen) at 200–400 nm are shown in Fig. 1F, and the maximal absorption peaks of collagen are both located in the interval 220–240 nm. This is due to the n→π∗ electron jumps of groups, such as C=O, -COOH and CO-NH_2_, which is consistent with the absorption properties of collagen.

### 3.2 Collagen coating

**Fig. 2:**
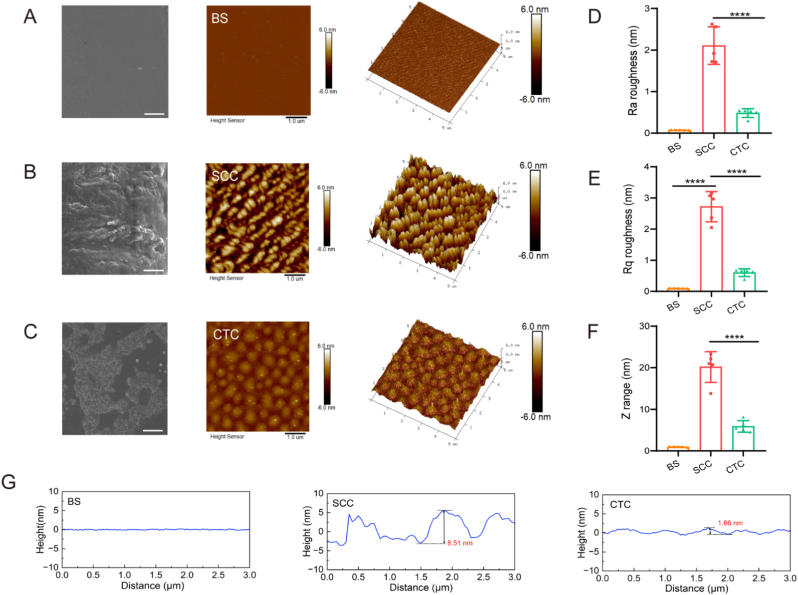
Construction of a collagen coating system. (A–C). SEM and AFM images (2D and 3D images) of BS, SCC and CTC collagen coatings. (D–F). Surface roughness parameters of the collagen coating. Linear roughness (Ra), root mean square roughness (Rq) and average peak and valley heights of the coating (Z range) were included. (G). Changes in height of coating surface. ∗∗∗∗ p < 0.0001.

To study the biological activity of SCC, several collagen coating systems were established for blank substrate (BS), SCC and CTC, respectively (Fig. 2A–C). Data showed that HUVEC cells adhered and grew effectively on both the SCC and CTC collagen coating, with live-dead staining revealing better biocompatibility than the control group (Fig. S1). AFM data (Fig. 2D–F) demonstrated that the surface roughness of SCC is significantly higher than that of the BS and CTC groups owing to an increase in the number of surface particles, which is consistent with the SEM results. The surface roughness was quantified in three ways: root-mean-square height (Rq), mean roughness (Ra) and Z-range. The Rq, Ra and Z-range of the SCC coatings were 2.7 ± 0.5 nm, 2.1 ± 0.5 nm and 20.2 ± 3.7 nm, respectively. The mean height difference of the SCC coating (8.5 nm) was also higher than that of the CTC coating (1.7 nm), and the roughness data all showed the same trend. The SCC coating exhibited the highest roughness.

### 3.3 SCC mediates macrophage polarization

**Fig. 3:**
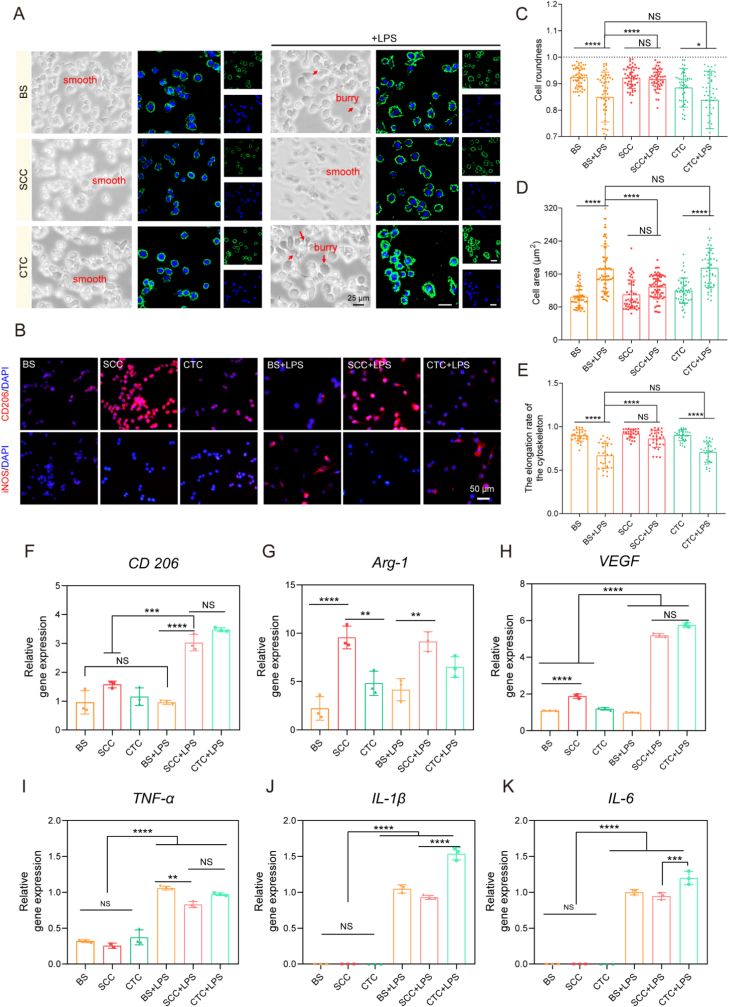
The regulation anti-inflammatory activity of collagen. (A). Morphological characterization and cytoskeleton diagram of RAW264.7 macrophages co-cultured with collagen coating for 6 h before and after LPS induction. (B). Staining of iNOS and CD206 on different collagen coatings in RAW264.7 macrophages before and after LPS induction for 24 h. (C–D). Cell roundness and cell area statistics calculated from Fig. 3A (n = 50). (E). Cytoskeleton elongation statistics calculated from Fig. 3A (n = 30). (F–H). M2 polarization-related and (I–K) M1 polarization-related gene expression in RAW 264.7 cells on the collagen coating treated with LPS (n = 3). NS no significant difference, ∗ p < 0.05, ∗∗ p < 0.01, ∗∗∗ p < 0.001, ∗∗∗∗ p < 0.0001.

In this study, the function of SCC in inflammatory regulation was examined, with particular emphasis on its impact on the behavior of macrophage polarization. A comparative analysis was conducted to assess the impact of collagen on the macrophage polarization of M1 to M2 morphology. The images of macrophages on various coatings were shown in Fig. 3A. The morphology of macrophages without LPS induction was found to be smooth and round, whereas after LPS-induced culture for 6 h, the roundness of macrophages on BS and CTC coatings changed more obviously than that of SCC coatings, and the cell area tended to become larger. Macrophages on SCC coatings recovered better roundness, and the cell areas did not change significantly. F-actin staining results were consistent with this finding (Fig. 3C–E). Macrophages are among the cells that interact with biomaterials and ultimately determine their fate. Features such as roughness of biomaterial surfaces also can activate macrophages, which in turn mediate into different phenotypes.

To further clarify the role of SCC on macrophage polarization, immunofluorescence staining showed that macrophages in the SCC coating group had the highest expression of CD206^+^M2 macrophages and low expression of iNOS^+^M1 macrophages compared to the blank substrate and CTC group (Fig. 3B). These findings indicated that SCC can drive M1 macrophages to decrease pro-inflammatory factor expression and increase anti-inflammatory factor expression, thus exerting an anti-inflammatory effect in the LPS-induced simulated inflammatory microenvironment. Consequently, SCC is postulated to have a regulatory role in inflammatory processes and the potential to effectively reverse and move in an anti-inflammatory direction. Gene expression analysis was also performed using qPCR (Fig. 3G–H), with *IL-6, IL-1β* and *TNF-α* identified as pro-inflammatory factor markers, *CD206, Arg-1* and *VEGF* selected as anti-inflammatory factor markers. Consequently, it indicates that SCC can mediate the polarization of M1 macrophages towards M2 macrophages, thereby contributing to the regulation of inflammatory processes and tissue regenerative remodeling.

**Fig. 4:**
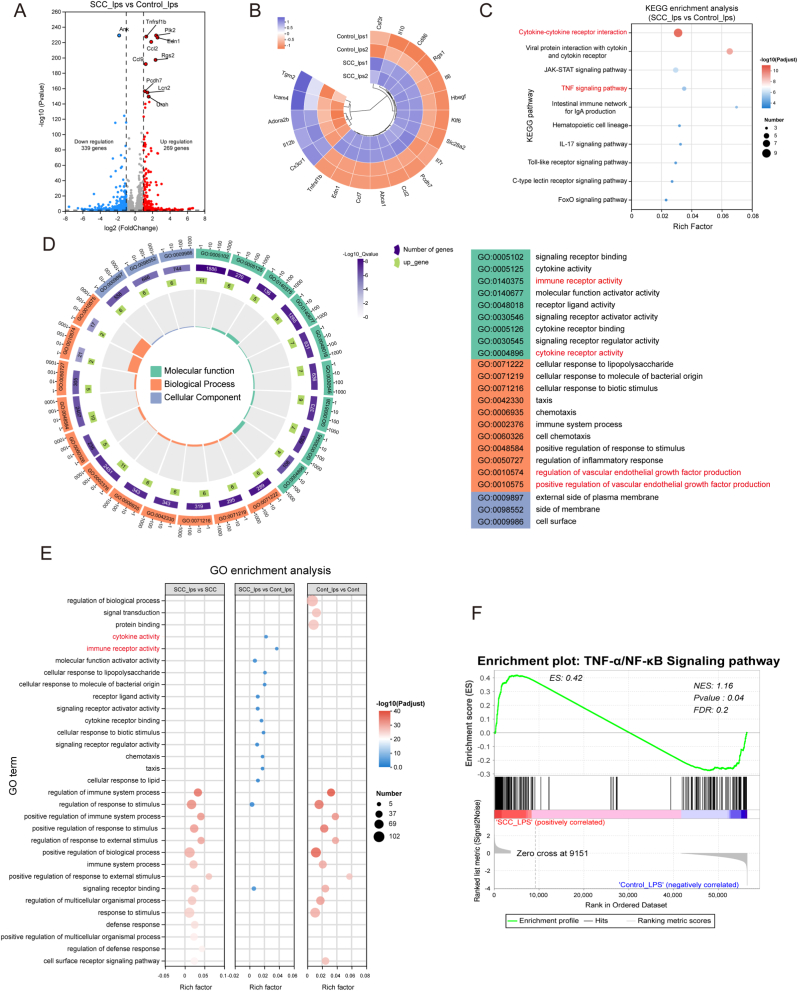
SCC mediates LPS-induced macrophage polarization into the M2 Phenotype. (A). RNA-Seq transcriptome analysis of the volcano map of differentially expressed genes (upregulated genes: red; downregulated genes: blue). (B). Heatmap of genes showing 20 regulated genes related to inflammation in the transcriptome analysis. Significant differential expression was determined using thresholds of p val ≤0.05 and ∣log2(Fold Change)∣ ≥ 1.0. (C). KEGG enrichment pathways in the SCC_lps group coincided with those in the control group. (D). GO enrichment analysis of biological process (BP), molecular function (MF) and cellular component (CC) in the SCC_lps group compared to the control group. (E). GO enrichment analysis of multiple gene sets. (F). GSEA enrichment analysis. (For interpretation of the references to colour in this figure legend, the reader is referred to the Web version of this article.)

The principal pathways responsible for the macrophage polarization from the M1-type to the M2-type include NF-κB, MAPK, PI3K/AKT and JAK-STAT [37,38]. To determine the potential mechanisms by which SCC modulate M1-type macrophages mediated into M2-type macrophages in the presence of LPS. Transcriptomic analysis was performed. Pearson’s correlation analysis and principal component analysis (PCA) revealed that the biological replicates within the two groups exhibited strong correlations and met the criteria for subsequent analysis (Fig. S2). A comparison of the SCC_lps and Control_lps groups revealed that 269 genes were upregulated and 339 genes were downregulated in the SCC_lps group (Fig. 4A). Furthermore, inflammation-related differentially expression genes (DEG) were analyzed, as shown in Fig. 4B. Genes associated with pro-inflammation (*Csf3r, Icam4, Il12b and Cx3cr1*) were significantly downregulated in the SCC_lps group, whereas genes associated with anti-inflammation (*Il10, Rgs1, Tnfrs1b and Abca1*) were significantly upregulated. GO (Gene Ontology) analysis of all differentially expressed genes (Fig. 4D) revealed their role in molecular functions (MF), such as immune receptor activity, cytokine receptor activity, and biological processes (BP), such as vascular endothelial growth factor production. These processes may be involved in inducing macrophage polarization from M1 to M2, thereby improving the local immune microenvironment. Furthermore, the results of the GO enrichment analysis of the multi-gene sets were the same (Fig. 4E). KEGG (Kyoto Encyclopedia of Genes and Genomes) enrichment analysis further mined the potential signaling pathways and found that inflammation-related signaling pathway TNF-α pathway, viral protein interaction with cytokines and cytokines receptor, and cytokine-cytokine receptor interaction were activated (Fig. 4C), which a similar outcome was demonstrated by GSEA (Gene Set Enrichment Analysis) (Fig. 4F). According to the analysis results of multiple gene sets using GSEA (Fig. S3), it has been proposed that activation of the TNF-α/NF-κB signaling pathway and its downstream channels.

### 3.4 Fabrication of PCL/SCC scaffold and biocompatibility

**Fig. 5:**
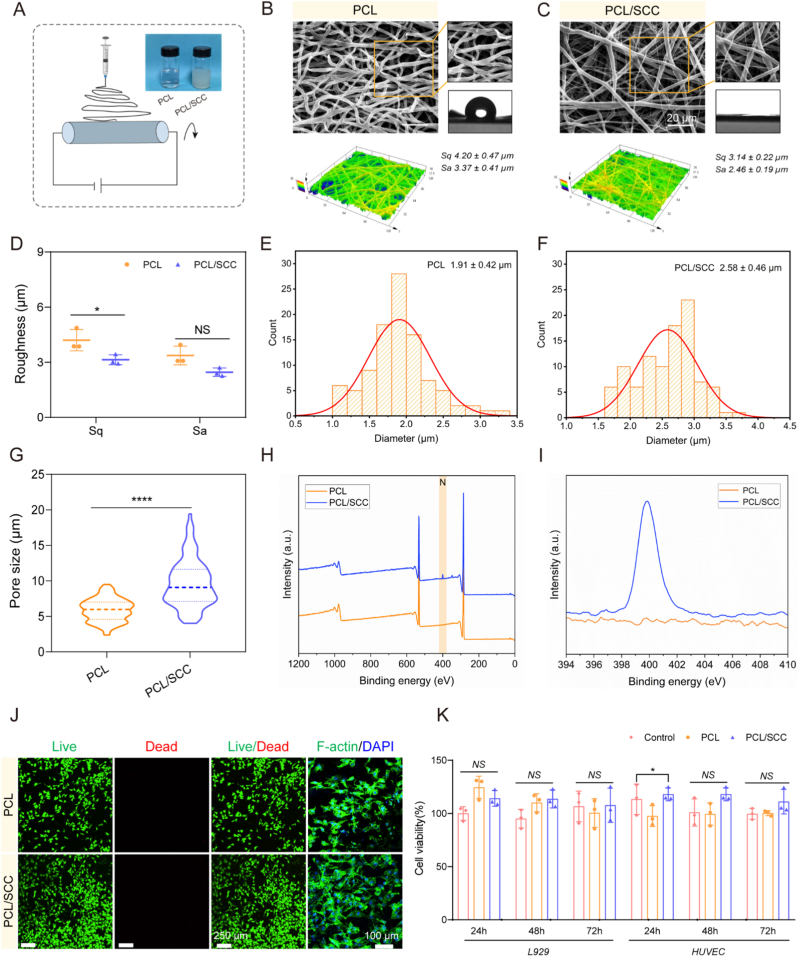
Biocompatibility evaluation of scaffolds. (A). Schematic of the scaffold prepared by electrospinning. (B–C). SEM images, contact angle tests and surface roughness characterization of PCL and PCL/SCC scaffolds. (D). Statistics of roughness data of scaffolds. Sq is the average height deviation of the surface roughness and Sa is the root mean square deviation of the surface roughness (n = 3). (E–F). Diameter distribution of fibrous scaffolds (n = 100). (G). Statistical plot of the pore size of the fiber scaffold (n = 100). (H–I). XPS wide scan spectra of PCL and PCL/SCC scaffolds with high-resolution N deconvolution spectra. (J). Live/dead staining (n = 3), HUVECs were co-cultured on the scaffolds for 24 h for F-actin staining. (K). Viability of L929 cells and HUVECs within 3 days (n = 3). NS no significant difference, ∗p < 0.05, ∗∗∗∗p < 0.0001.

We utilized electrospinning to prepare PCL/SCC membrane materials and small-diameter artificial blood vessels (Fig. 5A), in which the therapeutic effects of SCC were investigated. PCL and SCC were physically blended to formulate 15 % (w/v) PCL/SCC and PCL spinning solutions. Under the same parameter conditions, the membrane materials and small-diameter artificial blood vessels were obtained. The diameter of the vascular scaffold was 0.7 mm, and the thickness was 150 μm. The thickness of the membrane material was maintained at 250 μm for subsequent experiments. As shown in Fig. 5B–C, SEM images revealed the surface morphology of the fibers at the microscale. The morphology of each fiber was uniform and smooth. The contact angle test results indicated that the addition of SCC improved the wettability of the scaffold, and PCL/SCC scaffold exhibited hydrophilic properties. Compared with PCL, PCL/SCC scaffold showed an interlaced distribution of thick and thin fibers. We consider this is a special manifestation of the SCC component under the high-voltage effect of electrospinning. Overall, these fibers were randomly distributed to form an interlaced and interconnected three-dimensional porous structure. This not only enhanced the exchange and transportation of substances but also the high porosity provided sufficient three-dimensional space for cell growth and adhesion.

As shown in Fig. 5D, observations under the microscope revealed that the roughness (Sq = 4.2 ± 0.5 μm, Sa = 3.4 ± 0.4 μm) of the PCL scaffold was higher than that of the PCL/SCC (Sq = 3.1 ± 0.2 μm, Sa = 2.5 ± 0.2 μm). In the collagen-coated design (Fig. 2A–F), the SCC group showed a higher roughness compared to the CTC and BS groups. However, after blending into PCL (PCL/SCC group), it did not have an advantage in terms of roughness. Macrophages are the most studied immune cells in respect to the immunological response to biomaterials, and its activation and phenotype can be altered by biomaterial physicochemical characteristics such as surface topography, wettability and stiffness [39]. Hotchkiss KM et al. [40] studied the effect of surface modifications on macrophage activation and cytokine production under seven different cultivation conditions. Smooth Ti induced inflammatory macrophage (M1-like) activation, in contrast, hydrophilic rough titanium induced macrophage activation similar to the anti-inflammatory M2-like state. Moreover, macrophages play a key role in enhancing osseointegration of roughened Ti implants [41,42]. Roughened Ti causes anti-inflammatory macrophage polarization and increased production of TGF-β1, IL-10 and arginase. Therefore, surface characteristics such as roughness can have an impact on macrophage polarization. In this study, the differences in roughness were not very obvious in the spinning system. We consider that in the collagen coating system, the inflammatory regulation ability may be influenced by both the biological effect and the roughness. However, under the spinning condition, since the roughness difference of the scaffold was relatively small, we think it was the sole effect of the biological factor.

By randomly analyzing 100 fibers using Image J software, the fiber diameter of PCL was 1.9 ± 0.4 μm (Fig. 5E) and that of PCL/SCC was 2.6 ± 0.5 μm (Fig. 5F). It was found that the average pore diameters of PCL/SCC and PCL were 9.8 ± 3.7 μm and 5.9 ± 1.6 μm (Fig. 5G), respectively. These results indicated that the PCL/SCC scaffold was more appropriate for facilitating cell infiltration. Besides, the chemical functionality of the scaffolds was identified using XPS. There exist differences between PCL and PCL/SCC scaffold in the element composition from survey spectrum (Fig. 5H). Specifically, the appearance of the XPS characteristic peaks at 399.75 eV for N element was associated with the loading of SCC, which demonstrated the load efficiency of SCC (Fig. 5I). The roughness, hydrophilicity and hydrophobicity of the biomaterial surface are crucial for cell fate [43]. Consequently, a comprehensive analysis of parameters such as wettability, surface texture and charge are imperative for assessing the surface properties of biomaterials. It has been demonstrated that augmentation of the surface roughness of the material results in a substantial secretion of pro-inflammatory cytokines (e.g., IL-6 and TNF-α) by RAW264.7 macrophages in a time-dependent manner *in vitro* [44]. In addition, biocompatibility of the scaffolds was evaluated using live/dead staining, FITC staining and cell viability assays. It is generally accepted that materials obtained by blending polymer materials with natural biomaterials are nontoxic. However, some toxicity may be caused by organic solvent residues during the electrostatic spinning preparation process. As shown in Fig. 5J, after the cells contact the material, they first extend filopodia and expose integrins on their surfaces, which assists in the binding between cells and the material. Filopodia rich in F-actin can promote cell spreading on a material surface. It was found that more cells adhered to the PCL/SCC scaffold and the adhesion was closer. This indicated that the addition of natural biomaterials can improve cell compatibility, thus providing a more favorable microenvironment for cell growth. Subsequent CCK-8 assays on days 1, 2 and 3 showed over 90 % viability for both L929 cells and HUVECs. Based on biomaterial toxicity grades, both materials were nontoxic and cytocompatibility. The quantitative cytotoxicity results matched the live-dead staining results (Fig. 5K), further demonstrating the good biocompatibility of the PCL/SCC scaffold.

### 3.5 Hemocompatibility assessment of scaffolds

**Fig. 6:**
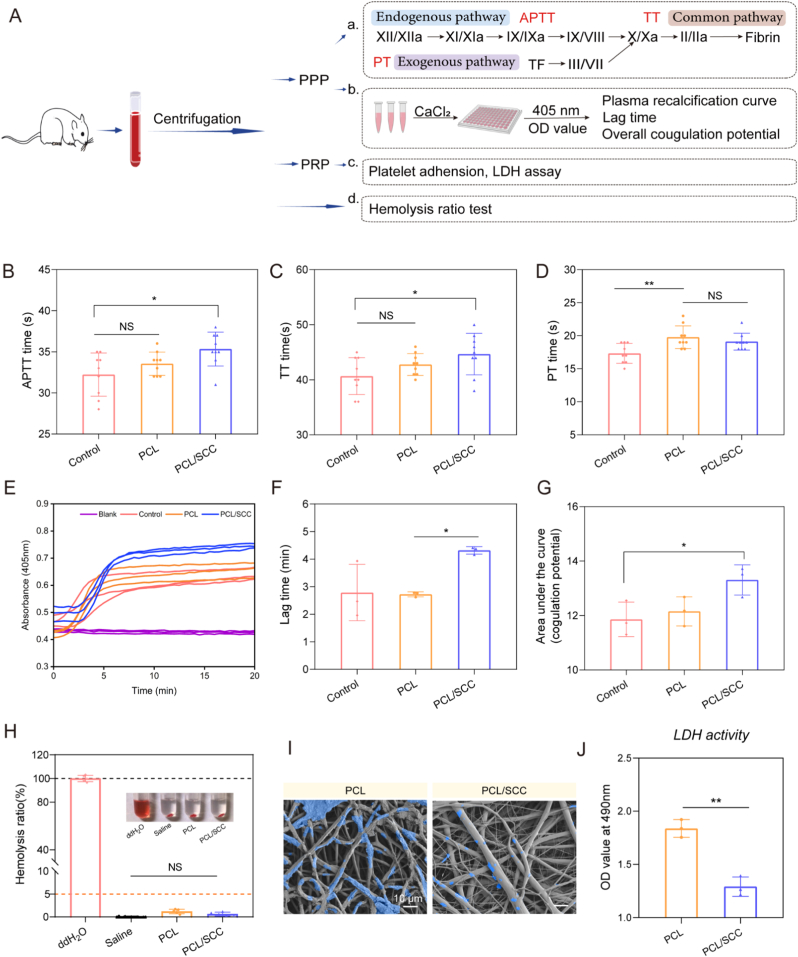
Hemocompatibility evaluation of scaffolds. (A). Schematic diagram of the blood-compatibility test. (B–D). APTT, TT and PT tests. APTT, activated partial thromboplastin time; TT, prothrombin time; PT, partial thromboplastin time. (E). Plasma recalcification curves (n = 3). (F). Clotting lag time. (G). Anticoagulant potential of the stent and quantitative results of the area under the curve. (H). Hemolysis rate tests (n = 5). (I). SEM images of platelet adhesion on the scaffolds. (J). LDH release assay (n = 3). ∗p < 0.05, ∗∗p < 0.01, ∗∗∗p < 0.001, ∗∗∗∗p < 0.0001.

Blood compatibility is crucial for clinical translation of biomaterials. Once implanted as foreign entities into the human body, biomaterials can promptly trigger blood coagulation upon blood contact, potentially leading to adverse events, such as thrombosis. Blood coagulation is an intricate cascade process in which coagulation factors are sequentially activated, ultimately converting fibrinogen into insoluble fibrin. The coagulation cascade pathway is typically categorized into three major branches: the intrinsic, extrinsic and common pathways (Fig. 6A). The intrinsic coagulation pathway is typically initiated when plasma-borne coagulation factors interact with negatively charged foreign surfaces. In contrast, the extrinsic pathway is triggered by exposure of tissue factor (TF), a molecule primarily derived from extravascular tissues, to the bloodstream, which commonly occurs during vascular injury. In essence, APTT reflects the integrity of the intrinsic and common coagulation pathways, PT assesses the extrinsic and common pathways, and TT evaluates the final common pathway of coagulation.

Quantitative analysis of the coagulation parameters revealed significant differences. The APTT results (Fig. 6B) demonstrated a longer APTT in the PCL/SCC group (35.3 ± 2.1 s) than in the PCL group. Similarly, the TT results (Fig. 6C) indicated that PCL/SCC (44.7 ± 2.0 s) exhibited a more favorable anticoagulant profile than PCL (42.8 ± 2.0 s). However, the PT results (Fig. 6D) showed comparable values between PCL/SCC (19.1 ± 1.3 s) and PCL (19.8 ± 1.7 s), suggesting no significant disparity in the extrinsic coagulation pathway. Overall, PCL/SCC significantly inhibited all three coagulation pathways compared to the control group, with a more pronounced inhibitory effect on the intrinsic and common pathways relative to the PCL group. Based on Fig. S4, SCC has advantages in this regard compared to other components. In the intrinsic coagulation pathway, plasma-derived coagulation factors bind to Ca^2+^ to form the prothrombin complex. This complex then activates prothrombin into thrombin, which in turn converts soluble fibrinogen into insoluble fibrin, culminating in blood clot formation. The plasma recalcification test, which involves re-introducing calcium into PPP to reinitiate the intrinsic coagulation process, serves as a straightforward *in vitro* assay for evaluating the thrombogenic potential of materials. As depicted in Fig. 6E, absorbance values were recorded at 405 nm every 30 s, and plasma recalcification curves were plotted using Image J software. Based on methodology proposed by J. J. Richards et al. [31] used the lag time, defined as the point at which the second derivative of the curve equals zero (f''(x) = 0), as a surrogate marker for the anticoagulant capacity of the materials. The results in Fig. 6F show that PCL/SCC had a significantly longer clotting initiation time (4.3 ± 0.1 min) than PCL (2.7 ± 0.1 min) and the control group (2.8 ± 1.0 min). In addition, following the approach reported by Zong et al. [32], the area under the plasma recalcification curve was calculated to predict the anticoagulant potential of the scaffolds. The data indicated that PCL/SCC (13.3 ± 0.5) outperformed PCL (12.2 ± 0.5), suggesting that the incorporation of SCC effectively enhances the anticoagulant properties of the material.

After co-culturing PRP with the scaffold, platelet adhesion on the sample surface was observed using SEM. The results are shown in Fig. 6I, many platelets adhered to the surface of the PCL, whereas only a few platelets adhered to the surface of the PCL/SCC group. As shown in Fig. 6J, we quantitatively analyzed this difference. According to the difference in OD values reflected by the lactate dehydrogenase (LDH) test results, it was further demonstrated that the number of platelets adhered in the control group was higher than that in the PCL/SCC group. In addition, both groups also maintained good indicators in terms of the hemolysis rate (Fig. 6H). In summary, PCL/SCC scaffold exhibits good antiplatelet adhesion properties and has the potential to improve the blood compatibility of blood-contacting biomaterials.

### 3.6 SCC incorporation PCL film promote M2 transition by *in vitro* and *in vivo* assays

**Fig. 7:**
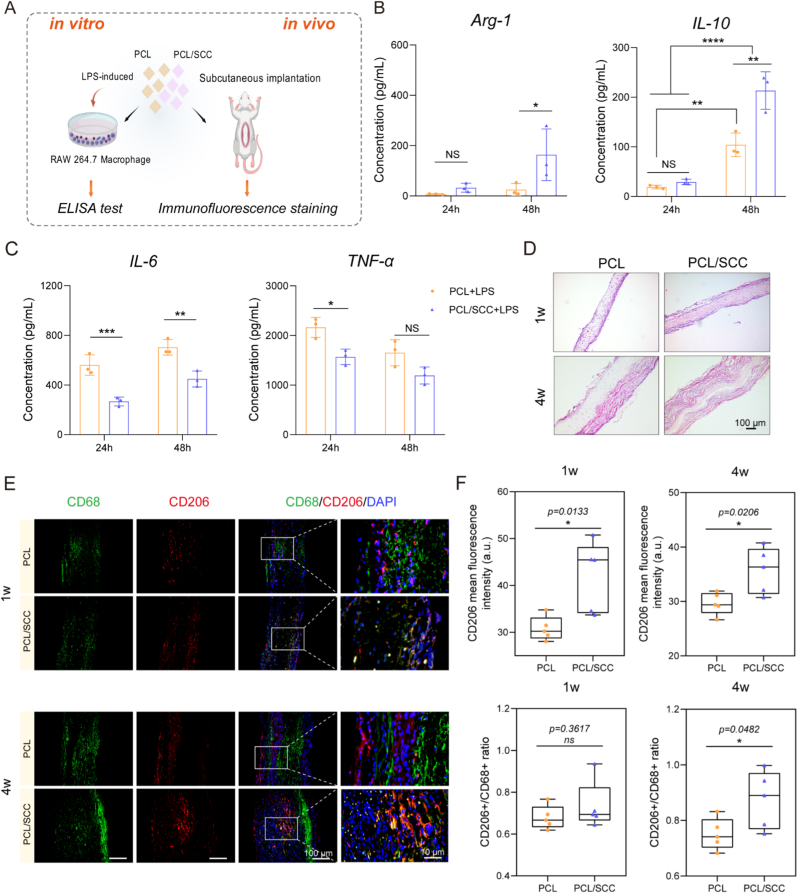
Histological analysis of scaffolds in a rat subcutaneous implantation model. (A). Schematic of scaffold inflammation assessment *in vitro* and *in vivo*. (B). ELISA results for IL-10 and Arg-1 as M2 subtype macrophage markers released by the scaffolds *in vitro* (n = 3). (C). ELISA results for IL-6 and TNF-α as M1 subtype macrophage markers released by the scaffolds *in vitro* (n = 3). (D). H&E staining in subcutaneously implanted samples at 1w and 4w. (E). Immunofluorescent staining of CD68 (green, whole macrophage) and CD206 (red, M2 macrophages). (F). Quantitative analysis of CD206 mean intensity and CD206^+^/CD68^+^ cells (n = 5). NS no significant difference, ∗ p < 0.05, ∗∗ p < 0.01. (For interpretation of the references to colour in this figure legend, the reader is referred to the Web version of this article.)

To determine whether the PCL/SCC scaffold with SCC could induce macrophage polarization and its therapeutic effects, we performed *in vitro* experiments (Fig. 7A). Under the condition of co-culture of cells and scaffolds in inflammatory microenvironment *in vitro*, the release level of M1 proinflammatory factors (such as IL-6 and TNF-α) in PCL/SCC scaffolds was lower than that the PCL scaffolds. At the same time, the PCL/SCC scaffold increased Arg-1 and IL-10 levels more than the PCL scaffold (Fig. 7B–C), suggesting that it can convert macrophages from M1 to M2 and reverse the inflammatory microenvironment. H&E staining (Fig. 7D) showed that the PCL/SCC scaffold had deeper cell infiltration after 4 weeks and was surrounded by a thicker and more uniformly infiltrated new tissue layer than the PCL scaffold.

Immunofluorescence staining was performed for *in vivo* macrophage polarization analysis. As shown in Fig. 7E and Fig. S5, we analyzed CD68 (whole macrophage), CD206 (M2 macrophages marker) and iNOS (M1 macrophages marker). The PCL/SCC scaffold had more CD206^+^ M2 macrophages and fewer iNOS^+^ M1 macrophages than the PCL scaffold did. Quantitative analysis revealed a higher M2/M1 ratio in the PCL/SCC group, and the CD206^+^/CD68^+^ ratio showed a similar trend (Fig. 7F). M2 macrophages help in tissue regeneration and fight inflammation, whereas M1 macrophages promote inflammation and hinder tissue repair. However, an excess of M1 may cause chronic inflammation and tissue necrosis, whereas an excess of M2 may encapsulate and diminish tissue function. Further research is therefore needed to optimize the M2/M1 balance and its relationship with the biomaterial-host response for better biomaterial design [19].

### 3.7 PCL/SCC reverses the inflammatory microenvironment in a mouse stiff artery transplantation model

**Fig. 8:**
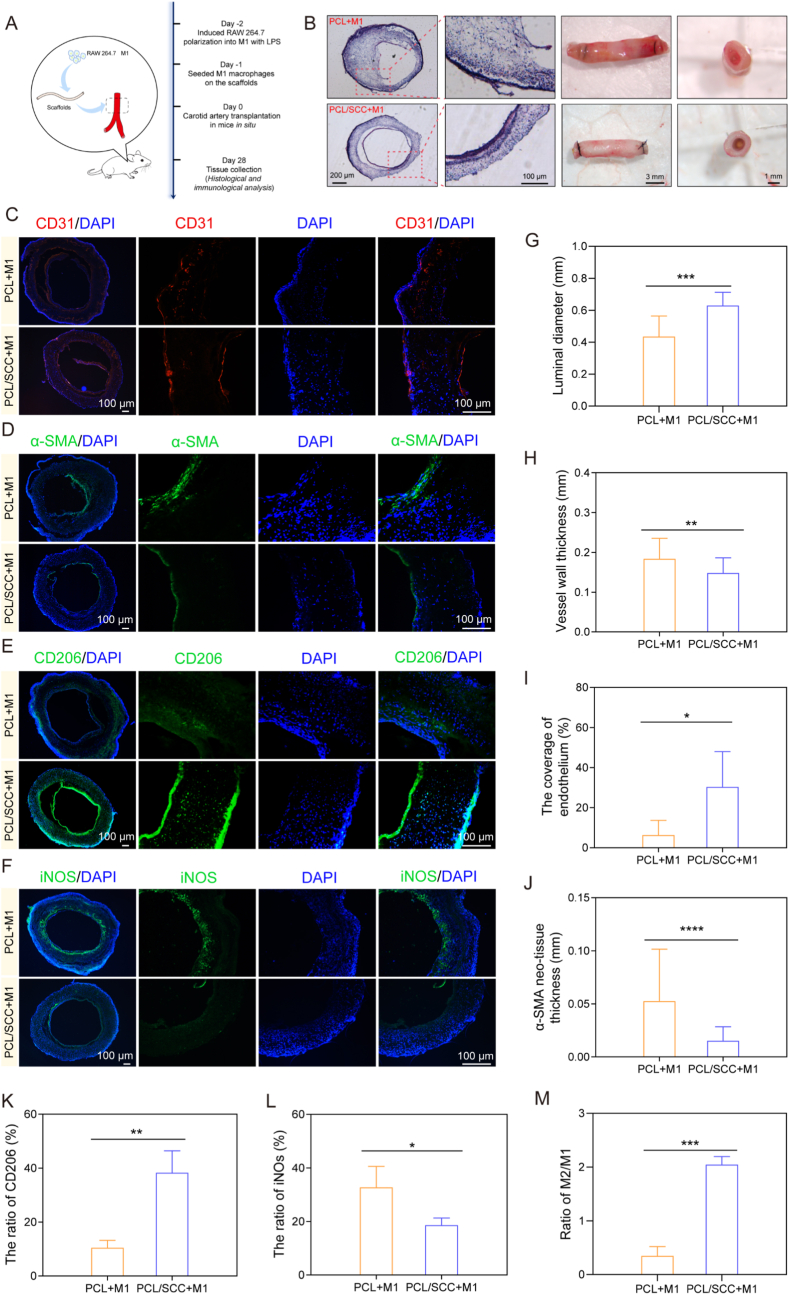
Histological analysis of mouse carotid artery grafts after 4 weeks. (A). Schematic diagram of carotid artery transplantation in mice. (B). Vessel surface morphology and hematoxylin and eosin staining. (C–F). Immunofluorescence staining of PCL and PCL/SCC scaffolds (n=3). (G–H). Quantitative H&E staining results, including vessel diameter and vessel wall thickness statistics. (I–J). Endothelial coverage and α-SMA neo-tissue thickness (n=3). (K–M). Quantitative statistical results for iNOS and CD206 expression (n=3). ∗ p < 0.05, ∗∗ p < 0.01, ∗∗∗ p < 0.001, ∗∗∗∗ p < 0.0001.

Furthermore, we pre-seeded M1 cells induced by LPS *in vitro* onto the vascular scaffold. We then used the mouse common carotid artery “Cuff” cannula model to orthotopically transplant the scaffold into the mouse carotid artery (Fig. 8A), to verify its effectiveness. One month after implantation, the blood vessels were harvested to observe patency. Histological and immunofluorescence staining were used to observe tissue regeneration and cell infiltration in blood vessels after a certain period of implantation. Statistics on the patency rate of transplanted blood vessels showed that the patency rate of the PCL/SCC group could reach 100 %, while that of the PCL group was only 33.3 %. The PCL group experienced severe blood vessel blockage (Supplementary Table S2). Combined with the previous characterization of blood compatibility, PCL/SCC exhibited excellent anticoagulant performance, which can effectively intervene in the blood coagulation process *in vivo*, thus promoting blood vessel patency. We believe that this is consistent with our expected hypothesis that the addition of SCC can improve the problem of blood vessel blockage. As shown in Fig. 8B, the results of H&E staining indicated that in the PCL/SCC+M1 group, cell infiltration was uniform, and the thickness and diameter of the blood vessel wall were stably maintained. Compared with the PCL/SCC+M1 group, the PCL+M1 group showed higher blood vessel thickness and smaller blood vessel diameter. The micro-blood vessels formed inside the scaffold can provide the transportation of oxygen and nutrients to new cells and promptly transport the generated metabolic waste, which is beneficial for tissue regeneration.

Rapid reendothelialization and early inhibition of smooth muscle hyperplasia are common requirements of most cardiovascular implants. CD31 staining showed that endothelial cells were distributed on the inner wall of the blood vessels (Fig. 8C). There was no significant difference between the groups, but the average endothelial cell coverage rate of PCL/SCC+M1 was higher than that of PCL+M1, indicating that the newly generated endothelial cells were more evenly distributed and more likely to form a complete endothelial layer. Severe hyperplasia of smooth muscle causes blood vessel blockage and poor patency. The results of α-SMA staining showed that the thickness of the vascular smooth muscle in the PCL+M1 group was significantly higher than that in the PCL/SCC+M1 group (Fig. 8D). PCL/SCC group promoted the formation of a complete endothelial layer on the inner side of the blood vessel wall, thus preventing thrombus formation and intimal hyperplasia and ensuring good blood vessel patency. CD206 staining showed that the number of CD206^+^ M2 macrophages in the PCL/SCC+M1 group was higher than that in the PCL+M1 group after 1 month (Fig. 8E). In contrast, iNOS staining indicated that the number of iNOS^+^M1 macrophages showed the opposite trend (Fig. 8F). Based on the quantitative results (Fig. 8G–M), indicating that the addition of SCC could polarize M1 macrophages into M2-type macrophages, promoting tissue repair and regeneration after the implantation. However, the inflammatory response after the implantation of artificial blood vessel stent material is a dynamic and evolving continuous process [45]. Optimizing the immune regulation of biological materials is not simply about pursuing “M2 polarization”, but lies in precisely guiding a dynamic, timely, moderate and self-limiting immune response program, which might be a more refined direction for regulating inflammation in the future. In summary, we believe that the PCL/SCC scaffold not only plays a promoting role in regulating the inflammatory microenvironment but also demonstrates certain advantages in the patency performance of the implanted blood vessels.

## 4 Conclusion

In this study, we examined the immunological impact of collagen extracted from fish swim bladders on macrophages under two distinct conditions: surface coating and incorporation into polymers. Our data proved that SCC could regulate macrophage polarization from M1 to M2 through the activation of TNF-α/NF-κB and its downstream signaling pathways. It dramatically improved the patency of PCL-based small-diameter vascular grafts by reversing the adverse inflammatory microenvironment *in vivo*, and thereby promoting tissue regeneration and remodeling. Overall, this new collagen derived from fish-swim bladders possesses the capacity to elicit a macrophage phenotype associated with anti-inflammatory resolution, thereby functioning as immunomodulatory agents and inducible materials of anti-inflammatory responses.

## CRediT authorship contribution statement

**Yuanchi Wang:** Writing – original draft, Investigation, Formal analysis, Data curation, Conceptualization. **Honghui Jiang:** Formal analysis, Conceptualization. **Yiping Wang:** Investigation, Formal analysis. **Yifan Wu:** Methodology, Investigation. **Xixi Wang:** Investigation, Formal analysis. **Ju Zhang:** Methodology, Investigation. **Yeqi Nian:** Investigation. **Jing Liu:** Writing – review & editing, Supervision, Resources, Project administration. **Zhihong Wang:** Writing – review & editing, Visualization, Supervision, Project administration.

## Declaration of competing interest

The authors declare that they have no known competing financial interests or personal relationships that could have appeared to influence the work reported in this paper.

## Data Availability

Data will be made available on request.
