# Supplementary material for: Distinctive fish collagen drives vascular regeneration by polarizing macrophages to M2 phenotype via TNF-α/NF-κB pathway
Source: Mater Today Bio. 2025 Sep 3;35:102273. doi: 10.1016/j.mtbio.2025.102273 (PMC12451383; doi:10.1016/j.mtbio.2025.102273)
Supplement: Multimedia component 1 [file mmc1.docx]

**Supporting Information**

Distinctive fish collagen drives vascular regeneration by polarizing macrophages to M2 phenotype via TNF-α/NF-κB pathway

Yuanchi Wang^1,2^, Honghui Jiang^1^, Yiping Wang^2^, Yifan Wu^3^, Xixi Wang^3^, Ju Zhang^1^, Yeqi Nian^2^, Jing Liu^1^*, Zhihong Wang^2^*

1 Tianjin Key Laboratory of Biomaterial Research, Institute of Biomedical Engineering, Chinese Academy of Medical Sciences and Peking Union Medical College, Tianjin 300192, China

2 Institute of Transplant Medicine, School of Medicine, Key Laboratory of Bioactive Materials of Ministry of Education, Nankai University, Tianjin 300071, China

3 College of Life Sciences, Tiangong University, Tianjin 300387, China

* Corresponding author

Prof. Zhihong Wang, nkwangzhihong@nankai.edu.cn

Prof. Jing Liu, liujing@bme.pumc.edu.cn


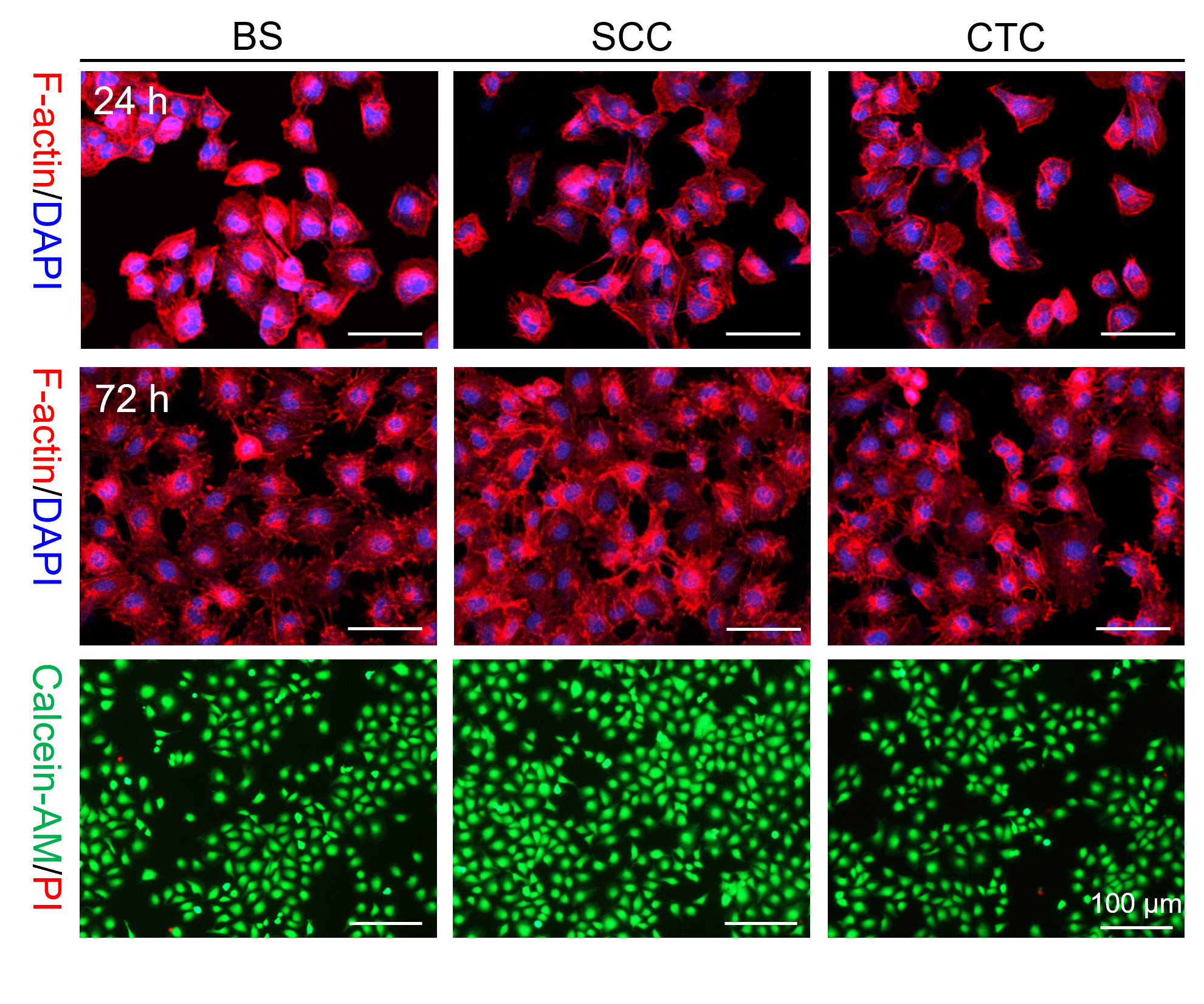


Fig. S1. Biocompatibility of collagen coatings. Live/dead staining and HUVEC cell adhesion on coating for 24 h and 72 h.


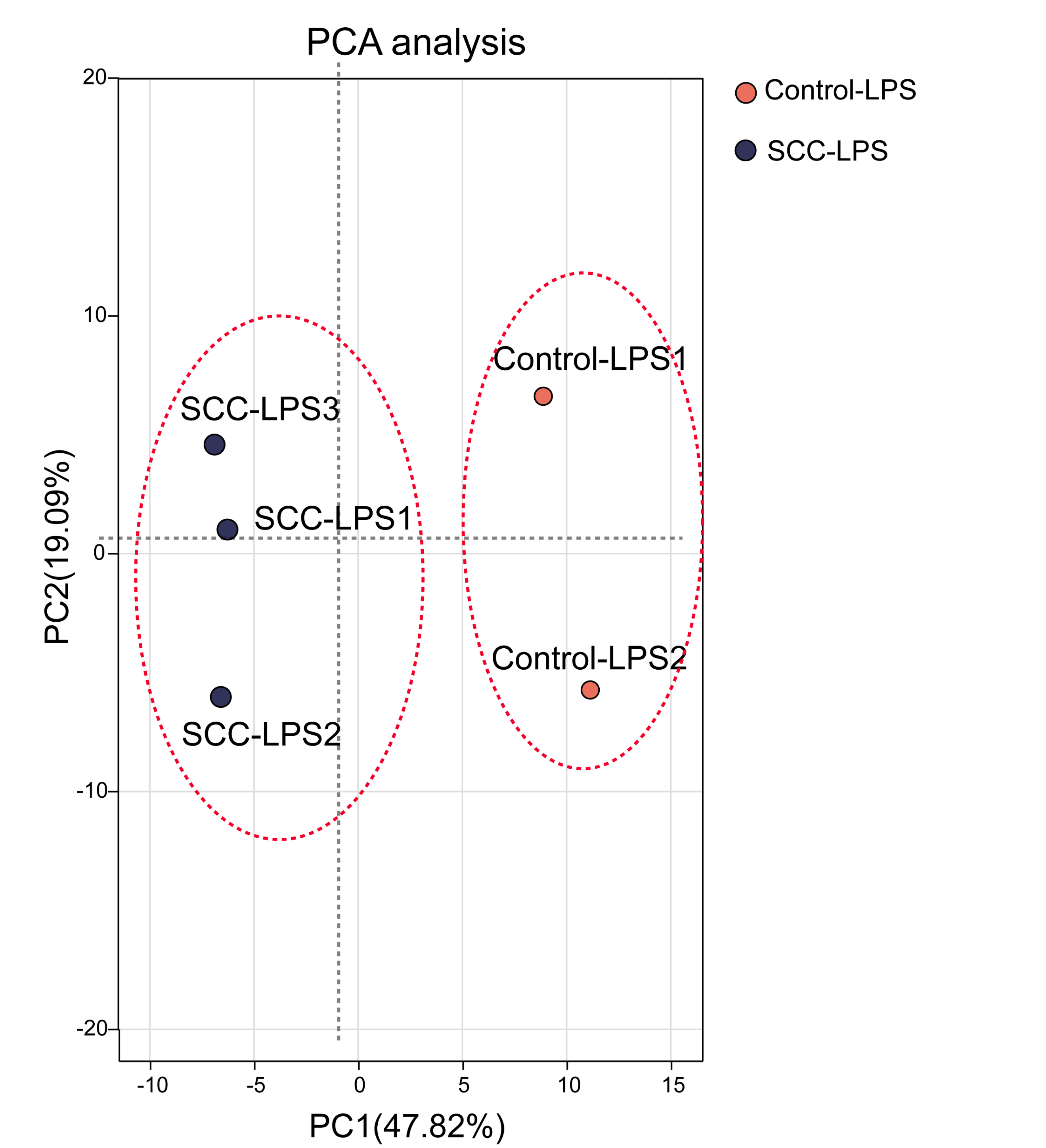

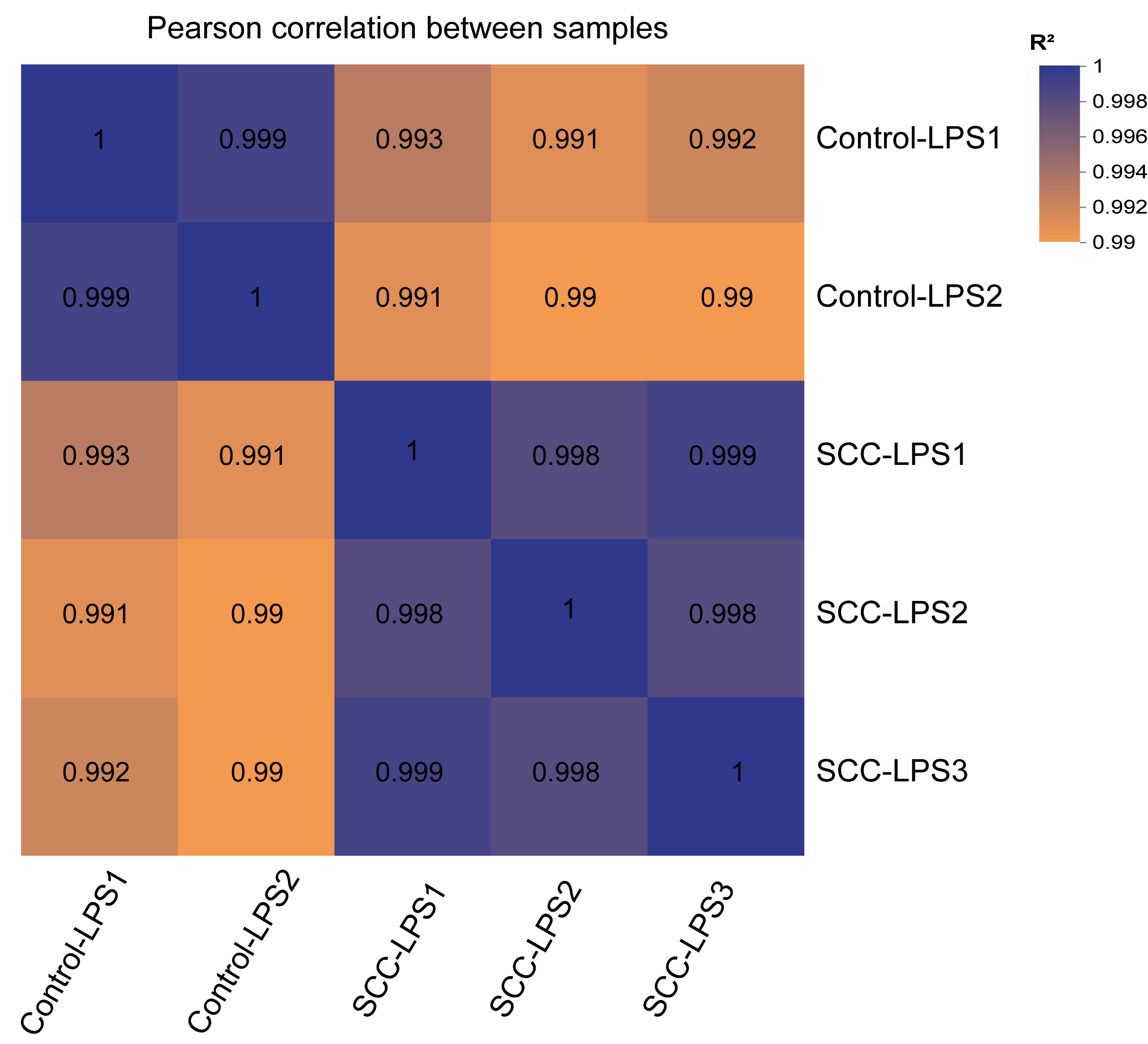


Fig. S2. PCA analysis and pearson’s correlation analysis of RNA sequencing results.


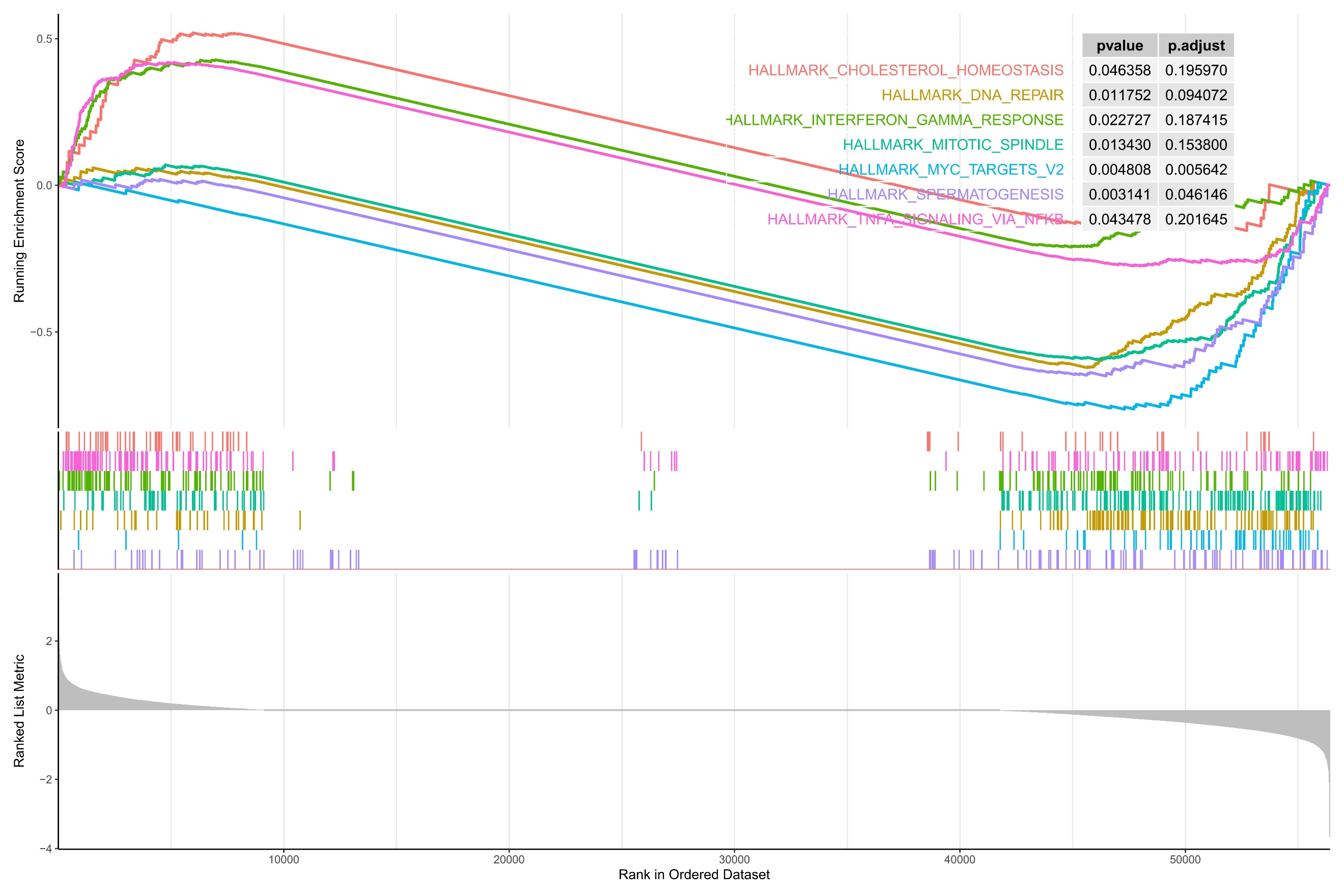


Fig. S3. Multi-pathway GSEA enrichment analysis.


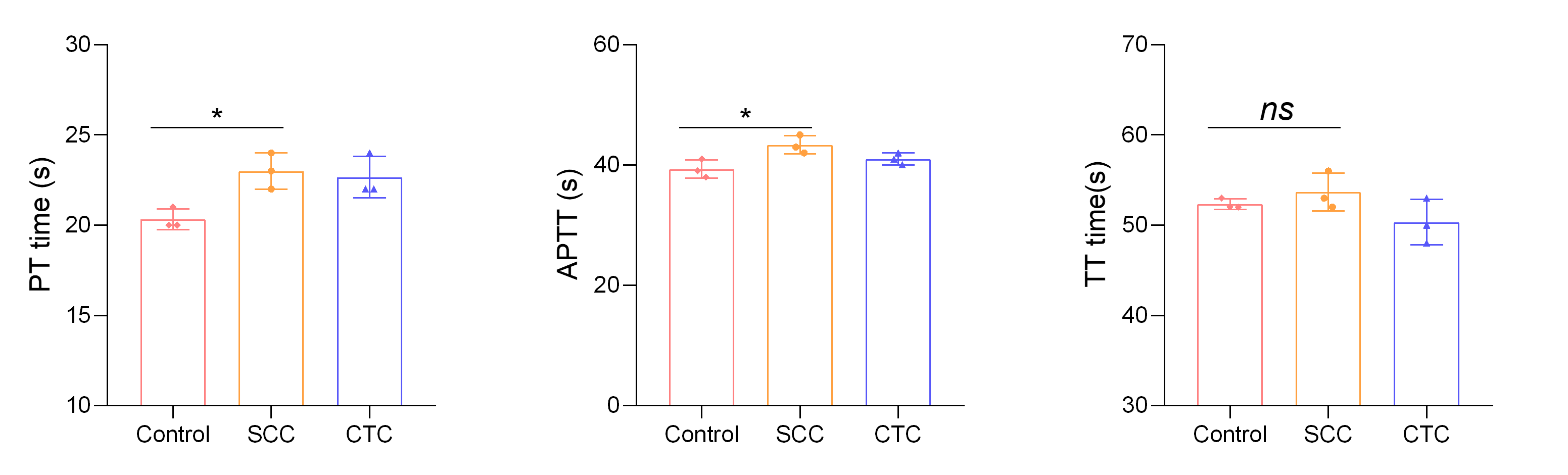


Fig. S4. Blood compatibility evaluation of collagen coating systems, including PT, APTT and TT.


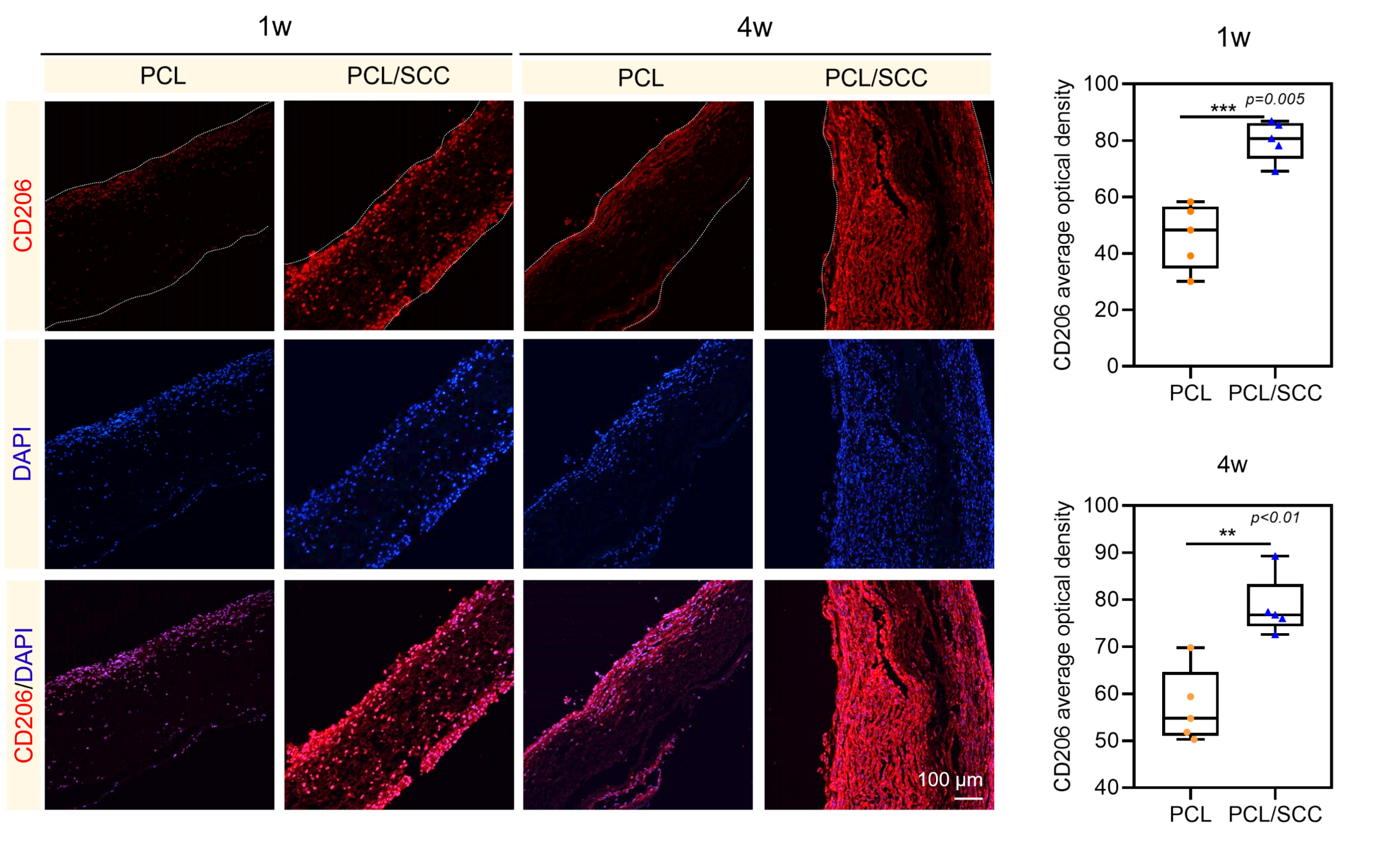


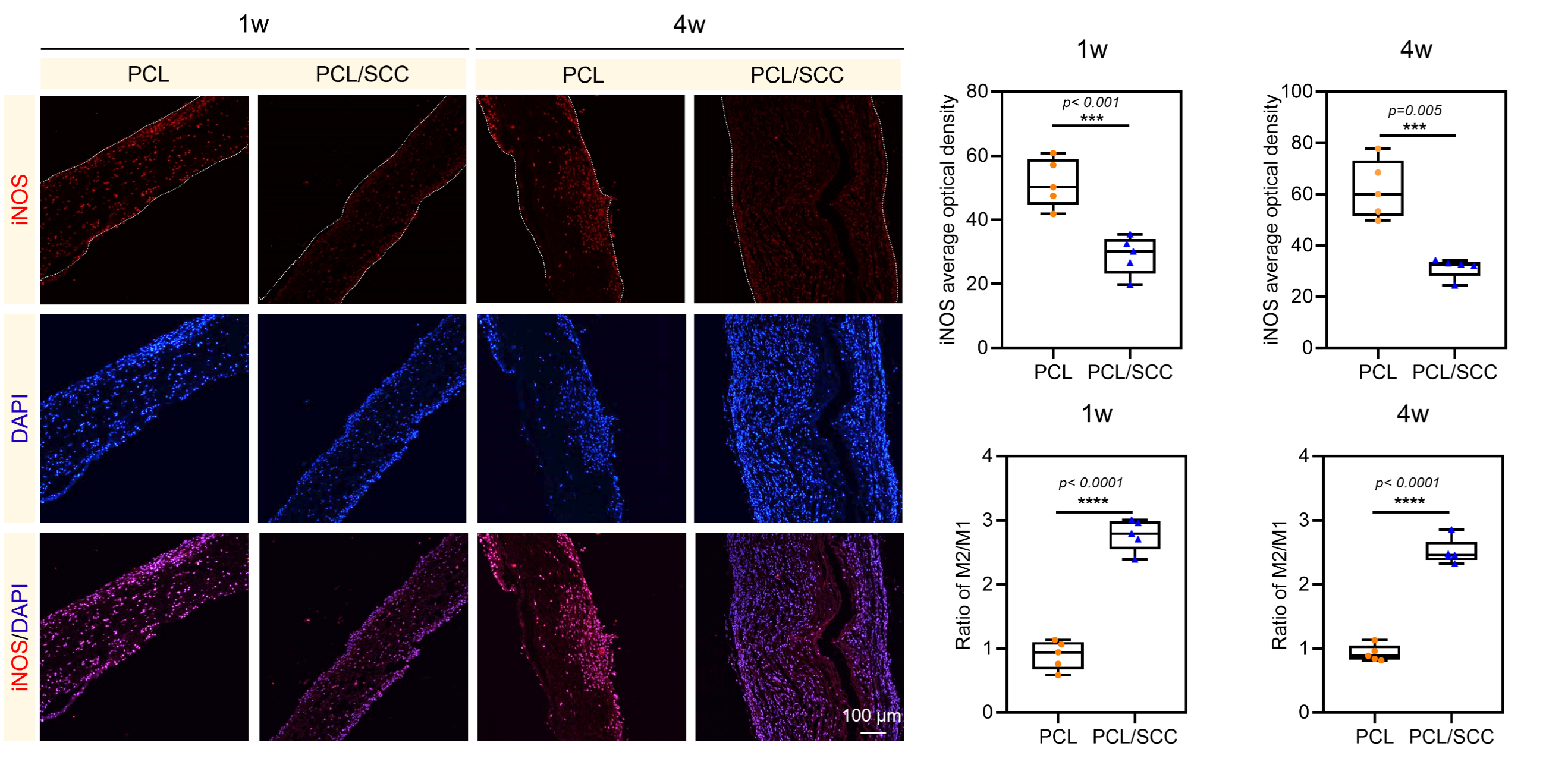


Fig. S5. Immunofluorescent staining of the subscutaneous implanted samples at 1w and 4w, and quantitative analysis of CD206, iNOS mean intensity and M2/M1 ratio (n=5).

Table S1. PCR primers for macrophages in this study

| Gene | Position | Primer sequences |
| --- | --- | --- |
| *Mouse-IL-6* | Forward | TACCACTTCACAAGTCGGAGGC |
|  | Reverse | CTGCAAGTGCATCATCGTTGTTC |
| *Mouse-TNF-α* | Forward | GGTGCCTATGTCTCAGCCTCTT |
| *Mouse-IL-1**β* | Reverse  Forward  Reverse | GCCATAGAACTGATGAGAGGGAG  ATCTCGCAGCAGCACATCAA  ATGGGAACGTCACACACCAG |
| *Mouse-Arg-1* | Forward | CATTGGCTTGCGAGACGTAGAC |
| *Mouse-CD206*  *Mouse-VEGF*  *Mouse-β-actin* | Reverse  Forward  Reverse  Forward  Reverse  Forward  Reverse | GCTGAAGGTCTCTTCCATCACC  GTTCACCTGGAGTGATGGTTCTC  AGGACATGCCAGGGTCACCTTT  CTGCTGTAACGATGAAGCCCTG  GCTGTAGGAAGCTCATCTCTCC  CATTGCTGACAGGATGCAGAAGG  TGCTGGAAGGTGGACAGTGAGG |

Table S2. Patency statistics of artificial blood vessel transplantation

| Group | Patency number |
| --- | --- |
| PCL+M1  PCL/SCC+M1 | 1/3  3/3 |
